# Priming with Recombinant Auxotrophic BCG Expressing HIV-1 Gag, RT and Gp120 and Boosting with Recombinant MVA Induces a Robust T Cell Response in Mice

**DOI:** 10.1371/journal.pone.0071601

**Published:** 2013-08-20

**Authors:** Rosamund Chapman, Helen Stutz, William Jacobs, Enid Shephard, Anna-Lise Williamson

**Affiliations:** 1 Institute of Infectious Disease and Molecular Medicine, Albert Einstein College of Medicine, Bronx, New York, United States of America; 2 Division of Medical Virology, Department of Clinical Laboratory Science, Albert Einstein College of Medicine, Bronx, New York, United States of America; 3 Department of Microbiology and Immunology, Albert Einstein College of Medicine, Bronx, New York, United States of America; 4 Department of Medicine, Faculty of Health Sciences, University of Cape Town, Cape Town, South Africa; 5 Medical Research Council, Cape Town, South Africa; 6 National Health Laboratory Service, Cape Town, South Africa; Federal University of São Paulo, Brazil

## Abstract

In previous studies we have shown that a pantothenate auxotroph of *Myocbacterium bovis* BCG (BCGΔ*panCD*) expressing HIV-1 subtype C Gag induced Gag-specific immune responses in mice and Chacma baboons after prime-boost immunization in combination with matched rMVA and VLP vaccines respectively. In this study recombinant BCG (rBCG) expressing HIV-1 subtype C reverse transcriptase and a truncated envelope were constructed using both the wild type BCG Pasteur strain as a vector and the pantothenate auxotroph. Mice were primed with rBCG expressing Gag and RT and boosted with a recombinant MVA, expressing a polyprotein of Gag, RT, Tat and Nef (SAAVI MVA-C). Priming with rBCGΔ*panCD* expressing Gag or RT rather than the wild type rBCG expressing Gag or RT resulted in higher frequencies of total HIV-specific CD8^+^ T cells and increased numbers of T cells specific to the subdominant Gag and RT epitopes. Increasing the dose of rBCG from 10^5^ cfu to 10^7^ cfu also led to an increase in the frequency of responses to subdominant HIV epitopes. A mix of the individual rBCGΔ*panCD* vaccines expressing either Gag, RT or the truncated Env primed the immune system for a boost with SAAVI MVA-C and generated five-fold higher numbers of HIV-specific IFN-γ-spot forming cells than mice primed with rBCGΔ*panCD* containing an empty vector control. Priming with the individual rBCGΔ*panCD* vaccines or the mix and boosting with SAAVI MVA-C also resulted in the generation of HIV-specific CD4^+^ and CD8^+^ T cells producing IFN-γ and TNF-α and CD4^+^ cells producing IL-2. The rBCG vaccines tested in this study were able to prime the immune system for a boost with rMVA expressing matching antigens, inducing robust, HIV-specific T cell responses to both dominant and subdominant epitopes in the individual proteins when used as individual vaccines or in a mix.

## Introduction

HIV infection is a major health problem in Southern Africa and, in particular, South Africa. Even though the infection rate appears to be stabilizing with the rollout of antiretroviral drugs, there are still an estimated 5.5 million people living with HIV in South Africa (11% of total population). The prevalence is higher (17.8%) amongst 15–49 year-olds (UNAIDS) and approximately 30% of women attending ante-natal clinics in South Africa were found to be HIV positive in 2010. The most effective way to control such an epidemic would be by means of vaccination.

The first indication that vaccination against HIV acquisition is possible has come from the encouraging outcome of the RV144 trial done in Thailand which has provided evidence that an HIV vaccine can decrease the rate of HIV infection. A combination of a canary poxvirus vector (ALVAC-HIV) expressing HIV-1 subtype B protease and Gag, and a fusion of subtype E and B envelope proteins with a bivalent envelope protein boost (subtype B and E; AIDSVAX B/E), was tested in the trial. The rate of HIV infection for participants vaccinated with the HIV vaccine combination as opposed to a placebo was 60% lower one year after vaccination and 31% lower at the end of three and a half years [1].

In this trial IgG binding antibodies to the variable regions 1 and 2 of the HIV-1 envelope protein were found to inversely correlate to the rate of HIV infection, whereas binding of plasma IgA antibodies to HIV-1 envelope directly correlated to the rate of HIV infection [2]. However, 31% efficacy is not sufficient to license a vaccine and thus a vaccine regimen with better protective efficacy is required. Elite controllers of HIV-1 have been shown to maintain highly polyfunctional, broadly directed central memory T cells that secret high levels of soluble mediators [3]. Thus a vaccine that induces this type of immune response should reduce HIV viraemia and prolong the time individuals take to progress to full blown AIDS. Maximum induction of HIV-specific T cell responses has been observed using heterologous prime-boost vaccine regimens where the vaccines used to prime and boost the immune system deliver matching antigens via a heterologous vector. Several studies in mice and non-human primates have indicated recombinant *Mycobacterium bovis* bacillus Calmette-Guérin (rBCG) expressing HIV antigens can efficiently prime the immune system for a heterologous boost with recombinant modified vaccinia virus Ankara (MVA), replication-deficient vaccinia virus (Dis), ovine atadenovirus or human adenovirus expressing HIV antigens [4]–[9].

In a previous study our group compared the use of wild type BCG Pasteur and a pantothenate auxotroph (Δ*panCD*) as vaccine vectors for HIV-1 subtype C Gag. When used in a prime-boost combination with a recombinant MVA expressing a matching Gag antigen, the rBCGΔ*panCD* expressing Gag (rBCGpan-Gag) primed higher Gag-specific CD8^+^ T cell responses than the wild type strain and protected against a surrogate vaccinia virus challenge [5]. In a further study we found that high levels of Gag-specific, polyfunctional CD8^+^ and CD4^+^ T cells and anti-Gag antibodies were induced in baboons primed with rBCGpan-Gag and boosted with Pr55 Gag virus-like particles [10].

Vaccine safety considerations, which are of especial importance when being used in immuno-compromised individuals, initiated the use of the pantothenate auxotroph strain of Pasteur. Replication of the BCG pantothenate auxtrophic strain in macrophages in the absence of pantothenate is very restricted and it is safer in SCID mice than the wild type strain [11]. Despite its limited replication *in vivo* a candidate TB vaccine, rBCG(Δ*panCD*)30 prepared using the BCG pantothenate auxtroph provided protection from challenge similar to that of the wild type strain of BCG in guinea pigs [11].

Until recently BCG vaccination was thought to protect against tuberculosis disease but not against infection. However, data generated using assays that distinguish between BCG vaccination and tuberculosis infection, have shown that BCG vaccination also protects against TB infection [12], [13]. A study carried out by the Pediatric Tuberculosis Network European Trials group comparing the tuberculin skin test to interferon-gamma release assays showed that BCG vaccination may reduce the risk of latent TB infection in children by more than 50% [14]. Thus development of BCG as a combined vaccine against TB and HIV could be of great benefit in preventing infants from becoming infected with TB or HIV.

As HIV-1 subtype C accounts for approximately 90% of infections in southern Africa, a vaccine targeting this subtype is required in South Africa. There is a need for an HIV vaccine to induce immune responses to epitopes in several HIV antigens to decrease the chance of immune escape. To address this plasmids that express either HIV-1 subtype C Gag, RT or Gp120 (truncated Env) were constructed and electroporated into *M. bovis* BCG Pasteur and the pantothenate auxotroph. The HIV genes selected for the vaccines were derived from two primary HIV-1 subtype C isolates, Du422 (*gag* and *rt* gene) and Du151 (*env* gene). These viral isolates have amino acid similarity to a derived South African consensus sequence [15]. Vaccines expressing HIV proteins based on a local consensus sequence are proposed to improve vaccine effectiveness by counteracting HIV genetic variability.

These recombinant BCG vaccines were tested individually in BALB/c mice for their ability to prime the immune system for a boost with a recombinant MVA, expressing a polyprotein of Gag, RT, Tat and Nef and a truncated Env (SAAVI MVA-C) [16]. Phase 1 Clinical Trials are currently being carried out using a matching DNA prime (SAAVI DNA-C) with a SAAVI MVA-C boost [17]. The magnitude of the response to subdominant and dominant epitopes in the expressed HIV protein were greater for the vaccines prepared with the pantothenate auxotroph of BCG than those vaccines prepared with the wild type. A mix of the individual vaccines vectored by the pantothenate auxotroph vector and expressing either Gag, RT or the truncated Env (Gp120) was found to prime the immune system for a boost with SAAVI MVA-C, which induced responses to subdominant and dominant epitopes in the three HIV proteins.

## Methods

### Construction of recombinant BCG (rBCG) and vaccine preparation

Wild type *M. bovis* BCG Pasteur 1172 P2 (BCG) (supplied by the Statens Seruminstitut, Denmark) and *M. bovis* BCG mc^2^6000 (BCGΔ*panCD*), a pantothenic acid auxotroph strain derived from BCG Pasteur (gift from W. R. Jacobs Jr., New York, USA), were grown on Middlebrook 7H10 agar supplemented with 10% oleic acid-albumin-dextrose-catalase and 0.5% glycerol (MB-7H10) or in Middlebrook 7H9 broth supplemented with 10% OADC, 0.2% glycerol and 0.025% tyloxapol (MB-7H9) on rollers (4 rpm) at 37°C. Kanamycin (10 μg/ml) was included in the media for plasmid selection where required. Media was supplemented with pantothenate (48 μg/ml) and hygromycin (50 μg/ml) for the growth of BCGΔ*panCD*.

The HIV-1 *gag*, *rt* and *env* genes used in this study were derived from those reported by Burgers *et* *al*., (2006) [18]. The 3′end of each HIV gene was fused to nucleotides encoding a 10 amino acid (RGPGRAFVTI) dominant BALB/c Env CD8^+^ T cell epitope within the V3 region of the HIV-1 subtype B envelope (peptide H) [19]. This allowed evaluation of the effect of the BCG vector on immune responses to subdominant and dominant HIV epitopes. The *Escherichia coli*/mycobacterial shuttle vector pEM19, that was used in this study, was created from pCB119 (gift from W. R. Jacobs Jr., New York) by deleting the *hsp60* promoter and *lys*A gene [5]. In this vector the HIV-1 gene of interest is fused to the nucleotides encoding the 19 kDa signal sequence and placed under the control of the *mtrA* promoter. The *mtrA* promoter is induced upon uptake of the rBCG by macrophages [20].

The shuttle vector pHS400 [5] contained the full length HIV-1 subtype C *gag* gene, codon optimised for use in BCG. However, analysis of this gene sequence revealed the existence of a number of potential internal ribosome binding sites and start codons with the potential to cause expression of out of frame nonsense proteins which would place an unnecessary metabolic burden on the mycobacterium and possibly compromise genetic stability. The *gag* gene was therefore re-codon optimised to remove these unfavourable sequence motifs, fused to the peptide H and cloned into the *Apa*I and *Hpa*I restriction sites of plasmid pHS400 [5] generating the shuttle vector pHS501. In all subsequent codon optimisations any potential ribosome binding site-like sequences were removed.

The BCG codon optimised *rt* gene was cloned between the *Apa*I and *Eco*RV restriction sites of pHS501, replacing the *gag* gene so that it was fused to the peptide H tag to create plasmid pRC501. The gp150 and gp120 (amino acids 1–723 and 1–488 respectively of Du151 Env, GenBank AF544008.1) portions of the *env* gene were codon optimised for expression in BCG, fused to the peptide H tag and cloned between the *Apa*I and *Hpa*I restriction sites of plasmid pHS400, creating plasmids pHS151 and pHS121 respectively.

The shuttle vectors pHS501, pRC501, pHS121, pHS151 and pCONEPI (vector which does not contain any HIV genes, Genbank accession DQ191755) were introduced into BCG and BCGΔ*panCD* by standard mycobacterial electroporation procedures [21]. All the rBCG vaccine stocks were prepared as described previously [5].

### Assessment of *in vitro* and *in vivo* genetic stability of the rBCG

To confirm *in vitro* genetic stability plasmid DNA was recovered from rBCG vaccine stocks and mapped with restriction enzymes. The HIV-1 genes from two plasmids per rBCG were also sequenced. *In vivo* stability was determined by the recovery of rBCG colonies from spleens of mice 6 weeks after immunization. Spleens were homogenized and plated on MB-7H10 medium containing the appropriate supplements. The plates were incubated at 37°C for 3–4 weeks and the rBCG colonies were then counted. To assess the genetic integrity of the rBCG as a measure of *in vivo* genetic stability, plasmid DNA was isolated from at least 40 colonies and the integrity determined using restriction enzyme digestion. The HIV genes of two plasmids from each rBCG were sequenced to confirm that no mutations had occurred.

### Mouse immunization

The vaccination schedule and all the procedures using female BALB/c mice (8–10 weeks old in groups of 5) were approved by the UCT Animal Ethics Committee (reference UCTAEC 07–107) and performed by a trained animal technologist. The individual rBCG vaccines or the rBCG-control vaccines were tested at doses of either 1×10^7^ cfu or 1×10^5^ cfu. A vaccine mix was prepared using 1×10^7^ cfu each of BCGΔ*panCD*[pHS501], (rBCGpan-Gag); BCGΔ*panCD*[pRC501], rBCGpan-RT; and BCGΔ*panCD*[pHS121] rBCGpan-gp120, then tested using a total dose of 3×10^7^ cfu. A dose of 3×10^7^ cfu of the rBCGpan-Control vaccine was used as the control for the vaccine mix. Mice were primed by intraperitoneal injection of 200 µl of the rBCG vaccine then boosted with rMVA [16] (10^4^ pfu/100 µl PBS), given on day 28 as an intramuscular injection, with 50 μl injected into the quadriceps muscles.

### Preparation of splenocytes for immune assays

Spleens were harvested and pooled from 5 mice per group 12 days after the rMVA booster vaccination on day 40. A single cell suspension of splenocytes was prepared then treated with erythrocyte lysing buffer (0.15 M NH_4_Cl, 10mM KHCO_3_, 0.1 mM Na_2_EDTA) for 1 min at room temperature before suspension in R10 culture medium (RPMI with 10% heat inactivated fetal calf serum (FCS) containing 15 mM β-mercaptoethanol, 100 U penicillin and 100 μg streptomycin/ml, (Invitrogen, Carlsbad, California, USA)).

### IFN-γ and IL-2 ELISPOT assays

The Mouse IFN-γ and IL-2 ELISPOT sets (BD Pharmingen, The Scientific Group, Johannesburg, SA) were used as per manufacturer's instructions. Splenocytes were plated at 1×10^5^/well in a 200 μl final volume of R10 alone (to determine background response) or medium containing 4ug/ml of an individual peptide (>95% HPLC pure, Bachem AG, Bubendorf, Switzerland) with amino acid sequence matching BALB/c CD8^+^ and CD4^+^ epitopes in Gag, RT and Env. For all the vaccines, responses to the dominant CD8^+^ T cell epitope tag (peptide H) were monitored using the peptide RGPGRAFVTI. Peptides with amino acid sequences AMQMLKDTI (Gag CD8^+^ peptide), NPPIPVGRIYKRWIILGLNK (Gag CD4^+^(13) peptide), FRDYVDRFFKTLRAEQATQE (Gag CD4^+^(17) peptide), VYYDPSKDLIA (RT CD8^+^ peptide), PKVKQWPLTEVKIKALTAI (RT CD4^+^ peptide), and YGVPVWREAKTTLFCA (Env CD4^+^(6) peptide) were included in the assays to monitor responses to subdominant epitopes in the respective HIV proteins [22]–[24]. Reactions were stopped after a 22 hour incubation at 37°C in 5% CO_2_, and spots were reacted with the detection antibody then developed with Nova Red as per the kit instructions. Spots were counted and analysed using an automatic ELISPOT reader (CTL technologies, Cleveland, Ohio) and Immunospot Version 3.2 software. Average spot numbers were calculated for triplicate reactions. For all experiments the coefficient of variation of the average (standard deviation (SD) of the average expressed as a percentage of the average spot numbers) was not more than 8%. Average spot numbers for responses to peptides that were twice that of average background spot numbers (absence of peptide) were considered positive. Values below this cut off were set to zero. Positive spot numbers were then adjusted to spot forming units (sfu) per 10^6^ splenocytes after background subtraction (not more than 30 sfu/10^6^ splenocytes). Cumulative IFN-γ ELISPOT responses were calculated as the sum of the responses to the individual subdominant Gag CD8^+^ and CD4^+^ peptides and dominant peptide H tag for the rBCG vaccines that expressed Gag or the sum of the responses to individual subdominant RT CD8^+^ and CD4^+^ peptides and dominant peptide H for the rBCG vaccines that expressed RT. When a mix of the individual rBCG vaccines was tested cumulative IFN-γ ELISPOT responses to Gag, RT and Env were calculated as the sum of the peptide responses to the individual CD8^+^ and CD4^+^ T cell epitopes in these proteins and the response to the dominant peptide H tag. All experiments were carried out on pooled spleens (n = 5) and in triplicate.

### Quantification of secreted cytokines

Splenocytes at a concentration of 7.5×10^6^ per ml R10 culture medium were cultured (48 h at 37°C in 5% CO_2_) in the absence of peptide (to detect background cytokine release) or with the individual peptides as used in the IFN-γ ELISPOT assay at 4 μg/ml. The cytokine content of culture supernatants was assayed using a cytokine bead array assay (BD Pharmingen, The Scientific Group, Johannesburg, SA) that detected IFN-γ, TNF-α, IL-6 and IL-10. The average of triplicate values was calculated and expressed as pg cytokine per 10^6^ splenocytes. The coefficient of variation of the average value (SD of the average expressed as a percentage of the average) was not more than 11%. Cytokine values were reported after background subtraction. For all mouse groups the average background cytokine release in the absence of peptide was not more than 3±1 pg per 10^6^ splenocytes. All experiments were carried out on pooled spleens (n = 5) and in triplicate.

### MHC class I peptide pentameric complex binding

APC-conjugated H-2K^d^ MHC class I pentameric complexes folded with the Gag peptide, AMQMLKDTI or the RT peptide VYYDPSKDLIA and H-2D^d^ MHC class I pentameric complexes folded with peptide H, RGPGRAFVTI were purchased from ProImmune (Oxford, UK). Aliquots of splenocytes (5×10^6^) pooled from groups of five mice were suspended in fluorescence-associated cell-sorting (FACS) buffer [phosphate-buffered saline (PBS) containing 1% FCS, and 0.02% NaN3] and labelled for 30 min at 4°C with the APC-conjugated pentameric complexes and PerCP-conjugated anti-CD8α (clone 53–6.7, eBiosciences, USA). Flow cytometric analysis was performed using a FACScalibur flow cytometer with Cell-Quest software (BD Biosciences). Pentamer-reactive cells were expressed as a percentage of gated CD8^+^ cells and presented as the average value of triplicates for a single experiment [25]. The coefficient of variation of the average value (SD of the average expressed as a percentage of the average) was less than 0.01%.

### Statistical analysis

Results are expressed as mean and standard deviation of the mean of the indicated number of experiments. Data was statistically analysed using Student's *t* test and *p* values of <0.05 were considered significant.

## Results

### Selection of genetically stable rBCG vaccines

*In vivo* and *in vitro* instability of recombinant BCG, especially those utilizing episomal vectors, is sometimes a problem. BCG[pHS501] (rBCG-Gag), BCGΔ*panCD*[pHS501] (rBCGpan-Gag), BCG[pRC501] (rBCG-RT) and BCGΔ*panCD*[pRC501] (rBCGpan-RT) were all found to be stable *in vitro*. However, no stable recombinants expressing the HIV-1 *gp150* gene could be obtained in either strain of BCG. *In vitro* stable recombinants of both strains of BCG expressing the truncated HIV-1 *gp120* gene were obtained (rBCG-gp120 and rBCGpan-gp120).

**Figure 1:**
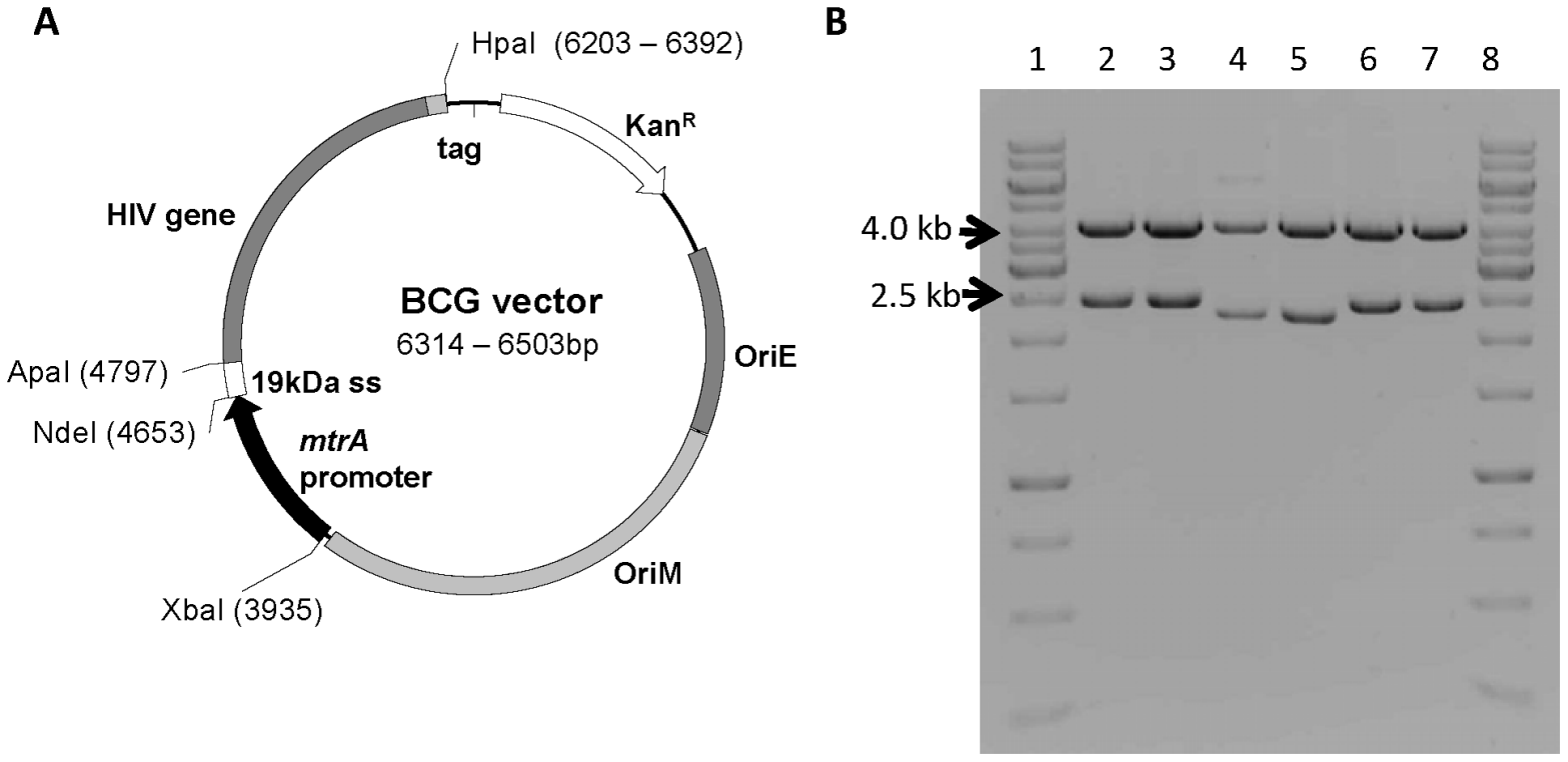
Schematic map and restriction enzyme digests of pHS501, pRC501 and pHS121 plasmid DNA isolated from rBCG. (**A**) Schematic map of *E. coli*/mycobacterial shuttle vector. (**B**) Lanes 1 & 8 contain the molecular weight marker GeneRuler^TM^ 1 kb Ladder (Fermentas, S.A.). Lanes 2 & 3 contain pHS501 plasmid DNA. Lanes 4 & 5 contain pRC501 plasmid DNA. Lanes 6 & 7 contain pHS121 plasmid DNA. Lanes 2, 4 & 6 contain plasmid DNA isolated prior to transformation into BCG (positive controls). Lanes 3, 5 & 7 contain plasmid DNA isolated from rBCG. Plasmid DNA in lanes 2 to 7 was digested with restriction enzymes *Xba*I and *Hpa*I. Results were the same for rBCG and rBCGΔ*panCD*. Enzymatic restriction analysis of only 2 representative samples are shown for each rBCG, however plasmid DNA was isolated from a minimum of 20 of each rBCG.

To assess *in vivo* stability rBCG-Gag, rBCGpan-Gag, rBCG-RT, rBCGpan-RT, rBCG-gp120, rBCGpan-gp120, rBCG-Control and rBCGpan-Control were isolated from the spleens of mice 6 weeks post vaccination and plated on MB-7H10 containing the appropriate supplements but lacking kanamycin. Plasmid DNA was isolated from at least 40 colonies for each recombinant and mapped with restriction enzymes. In addition the HIV genes were sequenced from 2 plasmids of each of the rBCG. All these recombinant BCG found to be stable *in vitro* remained stable *in vivo* (Figure 1).

### Use of a pantothenate auxotroph of BCG improves the priming ability of the rBCG vaccine

**Figure 2:**
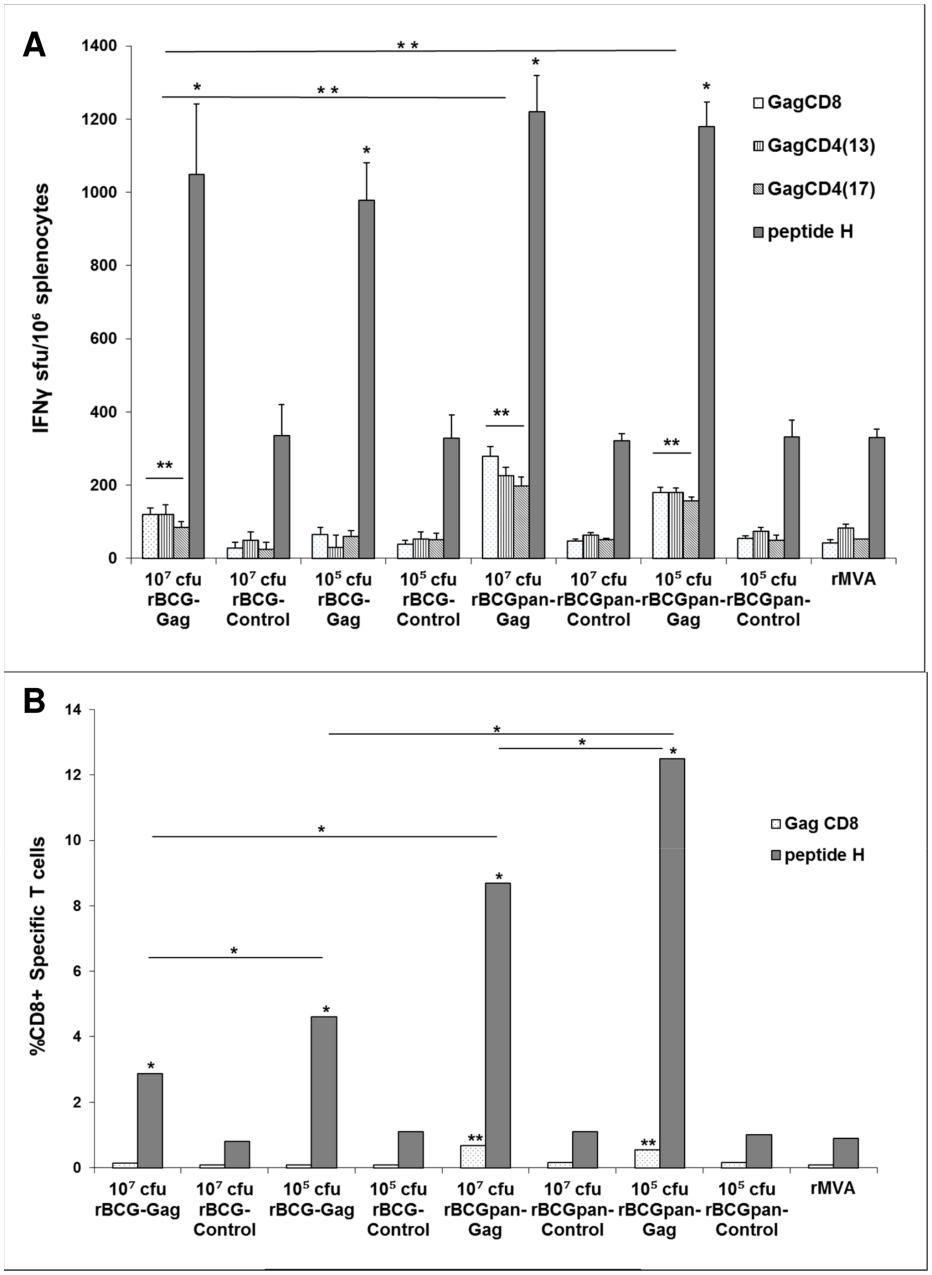
Influence of vector on IFN-γ ELISPOT responses to a rBCG-Gag or rBCGpan-Gag prime and rMVA boost. Groups of mice were primed with the indicated rBCG vaccines (10^7^ cfu or 10^5^ cfu) and then boosted with rMVA (10^4^ pfu) on day 28. One group of mice was left unvaccinated then vaccinated on day 28 with rMVA. On day 40 spleens were harvested and splenocytes pooled from 5 mice per group. (A) IFN-γ ELISPOT assays with Gag CD8^+^ T cells and CD4^+^ T cell peptides or peptide H CD8^+^ T cell peptide. Bars are the average and standard deviation of the average IFN-γ ELISPOT responses for the indicated individual peptides for 3 separate experiments. Asterisks indicate statistical significance of the mean IFN-γ ELISPOT responses for the individual peptides for a rBCG-Gag or rBCGpan-Gag vaccine prime and rMVA boost compared to that for the respective rBCG-Control or rBCGpan-Control vaccine prime and rMVA boost. (B) Splenocytes were stained with H-2K^d^ and H-2D^d^ MHC class I pentamers folded with the Gag CD8^+^ T cell peptide or peptide H CD8^+^ T cell peptide and flow cytometry was used to determine the frequency of Gag- and peptide H-specific CD8^+^ T cells in the splenocyte population. Bars are the average of triplicate values for Gag- and peptide H-specific CD8^+^ T cells expressed as a percentage of the total gated CD8^+^ T cell population for a single experiment. The coefficient of variation of all average values (standard deviation of the average expressed as a percentage of the average) was less than 0.01%. Asterisks indicate the statistical significance of the mean values for the percentage of Gag- or peptide H-specific CD8^+^ T cells for a rBCG-Gag or rBCGpan-Gag vaccine prime and rMVA boost compared to that for the respective rBCG-Control or BCGpan-Control vaccine prime and rMVA boost. Respective differences for peptide responses between groups are also indicated. *<0.01; **<0.05; Student's t-test for means of unpaired data.

**Figure 3:**
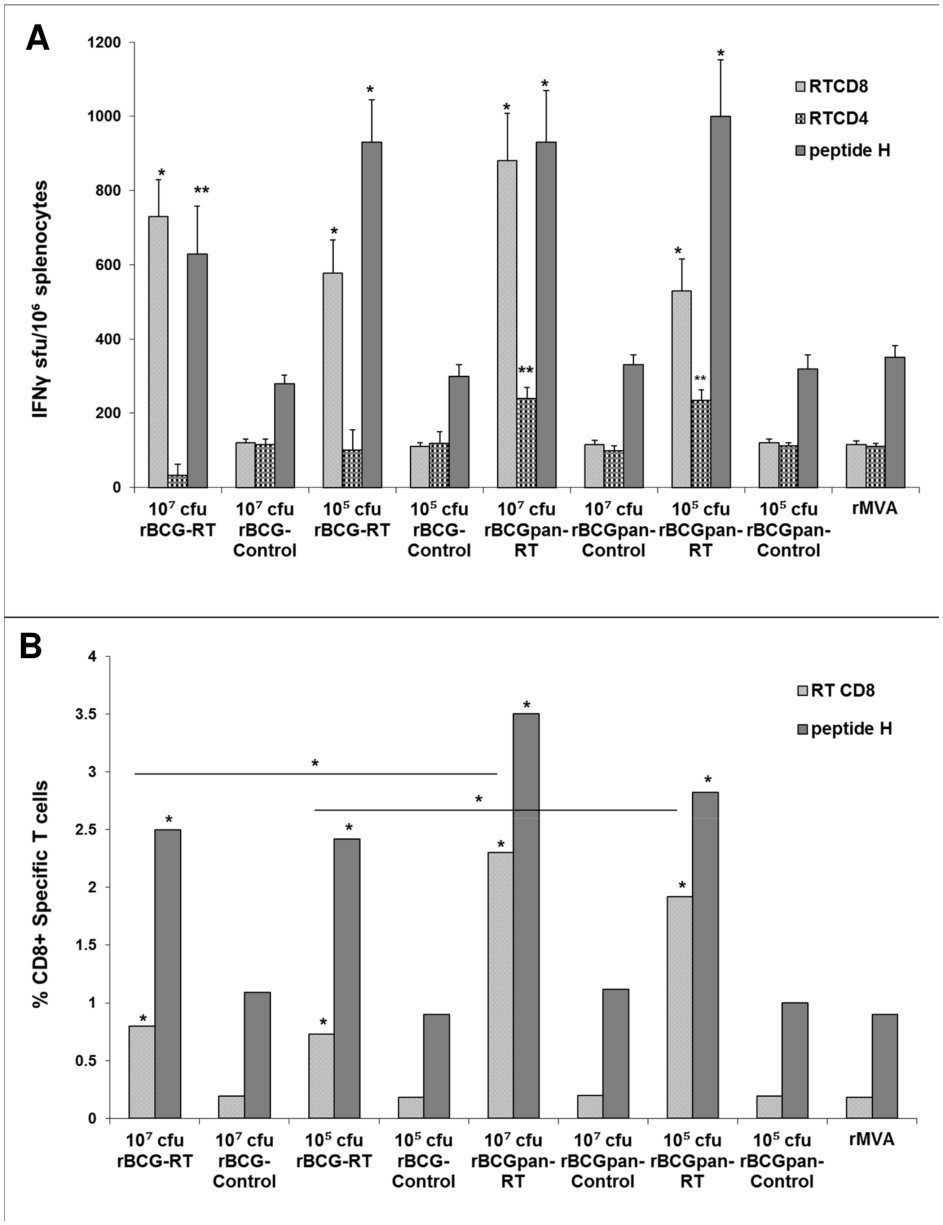
Influence of vector on IFN-γ ELISPOT responses to a rBCG-RT or rBCGpan-RT prime and rMVA boost. Groups of mice were primed with the indicated rBCG vaccines (10^7^ cfu or 10^5^ cfu) then boosted with rMVA (10^4^ pfu) on day 28. One group of mice was left unvaccinated then vaccinated on day 28 with rMVA. On day 40 spleens were harvested and splenocytes pooled from 5 mice per group. (A) IFN-γ ELISPOT assays with RT CD8^+^ T cell and CD4^+^ T cell peptides or peptide H CD8^+^ T cell peptide. Bars are the average and standard deviation of the average IFN-γ ELISPOT responses for the indicated individual peptides for 3 separate experiments. Asterisks indicate statistical significance of the mean IFN-γ ELISPOT responses for the individual peptides for a rBCG-RT or rBCGpan-RT vaccine prime and rMVA boost compared to that for the respective BCG- Control or rBCGpan-Control vaccine prime and rMVA boost. (B) Splenocytes were stained with H-2K^d^ and H-2D^d^ MHC class I pentamers folded with the RT CD8^+^ T cell peptide or peptide H CD8^+^ T cell peptide and flow cytometry was used to determine the frequency of RT- and peptide H-specific CD8^+^ T cells in the splenocyte population. Bars are the average of triplicate values for RT- and peptide H-specific CD8^+^ T cells expressed as a percentage of the total gated CD8^+^ T cell population for a single experiment. The coefficient of variation of all average values (standard deviation of the average expressed as a percentage of the average) was less than 0.01%. Asterisks indicate the statistical significance of the mean values for the percentage of CD8^+^ RT- or peptide H-specific T cells for a rBCG-RT or rBCGpan-RT vaccine prime and rMVA boost compared to that for the respective rBCG-Control or rBCGpan-Control vaccine prime and rMVA boost. Respective differences for peptide responses between groups are also indicated. *<0.01; **<0.05; Student's t-test for means of unpaired data.

The influence of BCG or BCGΔ*panCD* vectors as well as rBCG dose on immune responses to the HIV insert was evaluated by priming mice with doses of 10^7^ cfu or 10^5^ cfu of rBCG-Gag, rBCGpan-Gag, rBCG-RT, rBCGpan-RT or the respective rBCG-Control or rBCGpan-Control vaccine followed by a rMVA (10^4^ pfu) boost on day 28. Responses to subdominant Gag and RT CD8^+^ and CD4^+^ T cell epitopes and the dominant peptide H CD8^+^ T cell epitope tag were assayed on day 40 using the respective peptides in an IFN-γ ELISPOT assay (Figure 2 and Figure 3). In this study the dominant (as opposed to a subdominant) epitope response was considered to be one that provoked the most intense immune response.

Responses to Gag and RT CD8^+^ and CD4^+^ T cell peptides and the peptide H CD8^+^ T cell peptide for a rMVA vaccination alone were similar to that for a rBCG-Control or rBCGpan-Control prime (10^7^ cfu or 10^5^ cfu) and rMVA boost. Thus the vectors had no adjuvant activity (10^7^ cfu or 10^5^ cfu) or damping on HIV peptide responses (Figure 2 and Figure 3).

Similar peptide H-specific CD8^+^ T cell IFN-γ ELISPOT responses of 978 to1220 sfu/10^6^ splenocytes, that were 3-fold above control values (p<0.01), were observed in response to priming with either rBCG-Gag or rBCGpan-Gag (10^7^ cfu and 10^5^ cfu) (Figure 2A). Cumulative Gag-specific CD8^+^ T cell and CD4^+^ T cell IFN-γ ELISPOT responses, 3–4 fold above that of control values (p<0.05), of 324 sfu/10^6^ splenocytes and 516 to 701 sfu/10^6^ splenocytes, was achieved by priming with either rBCG-Gag (10^7^ cfu) or rBCGpan-Gag (10^5^ cfu and 10^7^ cfu) respectively. The cumulative Gag-specific IFN-γ ELISPOT responses induced by rBCGpan-Gag, although not influenced by dose, were significantly greater (p<0.05) from that induced by a prime with rBCG-Gag (10^7^ cfu) (Figure 2A). Subdominant Gag-specific responses contributed 24% or 35% to the total IFN-γ ELISPOT response for a rBCG-Gag (10^7^ cfu) prime or rBCGpan-Gag (10^7^ cfu or 10^5^ cfu) prime respectively (Figure 2A). Thus using BCGΔ*panCD* rather than BCG as the vector allows the induction of higher responses to subdominant Gag epitopes with the dose having minimal influence on the magnitude of the response to the Gag insert.

A single experiment was performed using H-2K^d^ and H-2D^d^ MHC class I pentamers folded with the Gag CD8^+^ T cell peptide or the peptide H CD8^+^ T cell peptide respectively to directly enumerate Gag- and peptide H-specific CD8^+^ T cells as a percentage of total gated CD8^+^ T cells (Figure 2B). Cumulative frequencies of Gag- and peptide H-specific CD8^+^ T cells of not more than 1.3% of the total CD8^+^ T cell population was induced by a rBCG-Control prime or rBCGpan-Control prime (10^7^ cfu or 10^5^ cfu) and rMVA boost which was not different from a rMVA vaccination alone (Figure 2B).

A rBCGpan-Gag (10^7^ cfu and 10^5^ cfu) prime induced more peptide H-specific CD8^+^ T cells than the respective rBCG-Gag prime (p<0.01). In addition, priming with rBCGpan-Gag resulted in higher cumulative frequencies of Gag- and peptide H-specific CD8^+^ T cells (9.4% and 13.1% of the total CD8^+^ T cells for a dose of 10^7^ cfu and 10^5^ cfu respectively) than rBCG-Gag (3% and 4.7% for a dose of 10^7^ cfu and 10^5^ cfu respectively). Priming with 10^5^ cfu of rBCG-Gag or rBCGpan-Gag induced significantly (p<0.01) higher peptide H-specific CD8+ T cells than doses of 10^7^ cfu (Figure 2B). Subdominant Gag-specific CD8^+^ T cells were only observed with a rBCGpan-Gag prime, these CD8+ T cells contributed 7.2% (10^7^ cfu) and 4.3% (10^5^ cfu) to the respective cumulative frequencies (Figure 2B).

The choice of vector for expression of RT did not influence RT- and peptide H-specific CD8^+^ T cell IFN-γ ELISPOT responses. Peptide H-specific CD8^+^ T cell IFN-γ ELISPOT magnitudes of 629 to 1000 sfu/10^6^ splenocytes, that were 3-fold above control values (p<0.01 or p<0.05), and RT-specific CD8^+^ T cell magnitudes of 530 to 730 sfu/10^6^ splenocytes that were 5-fold above control values (p<0.01), were observed after priming with either rBCG-RT or rBCGpan-RT (10^7^ cfu and 10^5^ cfu) (Figure 3A). No RT-specific CD4^+^ T cell peptide responses above control values were induced by a rBCG-RT prime. In contrast, priming with rBCGpan-RT (10^7^ cfu and 10^5^ cfu) induced similar RT CD4^+^ T cell IFN-γ ELISPOT responses of 235 to 240 sfu/10^6^ splenocytes that were 2-fold above control values (p<0.05). Thus the advantage of priming with BCGpan-RT as opposed to rBCG-RT prior to the rMVA boost is the induction of subdominant RT CD4^+^ T cell peptide responses.

A further single experiment using H-2K^d^ and H-2D^d^ MHC class I pentamers folded with the RT CD8^+^ T cell peptide or the peptide H CD8^+^ T cell peptide respectively was performed to directly enumerate RT CD8^+^ T cell and peptide H CD8^+^ T cell frequencies as a percentage of total gated CD8^+^ T cells (Figure 3B). Cumulative RT- and peptide H-specific CD8^+^ T cell frequencies induced by either a rBCG-Control or rBCGpan-Control prime (10^7^ cfu or 10^5^ cfu) and rMVA boost or rMVA vaccination alone were not significantly different and were not more than 1.3% of the total CD8^+^ T cell population (Figure 3B). The choice of vector (either BCG or BCGΔ*panCD*) for RT did not influence the frequency of peptide H-specific CD8^+^ T cells. However RT-specific CD8^+^ T cell frequencies induced by priming with rBCGpan-RT prime were significantly higher (p<0.01) than for a respective rBCG-RT prime. Cumulative RT- and peptide H-specific CD8^+^ T cell frequencies reached approximately 3.3% of the total CD8^+^ cells for a rBCG-RT (10^7^ cfu or 10^5^ cfu) prime with 23% of the response from responding RT-specific CD8^+^ T cells (Figure 3B). In contrast for a rBCGpan-RT (10^7^ cfu or 10^5^ cfu) prime cumulative RT- and peptide H-specific CD8^+^ T cell frequencies reached approximately 5.0% of total CD8^+^ T cells with 40% of the response being from RT-specific CD8^+^ T cells (Figure 3B). Thus using BCGΔ*panCD* as a vector rather than BCG for RT expression is advantageous in influencing the level of subdominant RT-specific CD8^+^ T cell frequencies. The priming dose did not significantly influence the magnitude of the response (Figure 3B).

### Immune responses to HIV-1 Gag, RT and Env can be achieved when a mixture of the rBCGΔ*panCD* vaccines are used

The need for an HIV vaccine to induce responses to several HIV antigens led us to investigate induction of immune responses in mice to a prime with a mix of rBCG vaccines each expressing Gag, RT or Gp120. The BCGΔ*panCD* vector rather than the wild type BCG vector was used for these studies as we found it primed responses to subdominant HIV epitopes (see previous section).

A dose of 10^7^ cfu of each individual vaccine was mixed to provide a vaccine mix with a dose of 3×10^7^ cfu in the injection volume. Mice were primed with this dose of vaccine mix then boosted with rMVA (10^4^ pfu) on day 28. The experiment also included a prime with each of the individual vaccines (10^7^ cfu) and rMVA boost on day 28 for comparison purposes. Priming with the rBCGpan-Control (10^7^ cfu or 3×10^7^ cfu) followed by a rMVA (10^4^ pfu) boost on day 28 was used as a control. Splenocytes were collected on day 40 and used in IFN-γ and IL-2 ELISPOT assays with peptide H, the CD8^+^ T cell epitope tag on each HIV protein, and peptides to CD8^+^ T and CD4^+^ T cell epitopes in Gag, RT and Gp120 to determine the immune response. The results shown are from three separate experiments.

Cumulative IFN-γ (503±15 sfu/10^6^ splenocytes) and IL-2 ELISPOT (108±18 sfu/10^6^ splenocytes) peptide responses for a rBCGpan-Control (3×10^7^ cfu) prime and rMVA boost were not significantly different from cumulative IFN-γ (597±35 sfu/10^6^ splenocytes) and IL-2 (98±19 sfu/10^6^ splenocytes) ELISPOT responses for a rMVA vaccination alone. Thus using a dose of 3×10^7^ cfu did not induce any adverse effect on HIV peptide responses in the ELISPOT assays.

**Figure 4:**
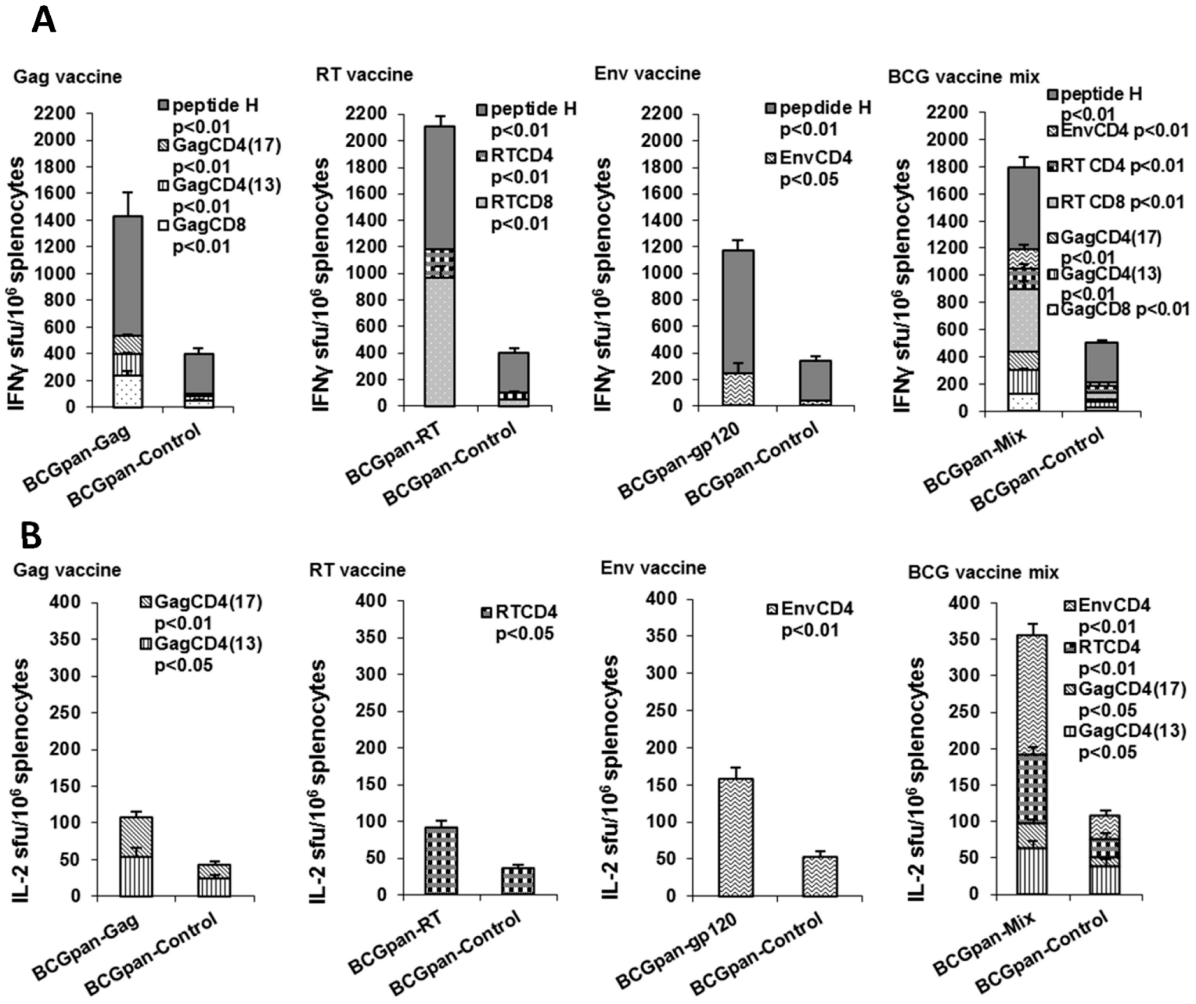
IFN-γ ELISPOT responses induced by a prime with the individual rBCG-HIV vaccines or a mix of the rBCG-HIV vaccines and rMVA boost. Groups of mice were primed with either the individual rBCGpan-Gag, rBCGpan-RT or rBCGpan-gp120 vaccines (10^7^ cfu) or a rBCG vaccine mix (3×10^7^ cfu) prepared by mixing 10^7^ cfu of the individual rBCGpan-Gag, rBCGpan-RT and rBCGpan-gp120 vaccines. Groups of mice vaccinated with the rBCGpan-Control vaccine (10^7^ cfu to act as control for the individual vaccine vaccinations or 3×10^7^ cfu to act as control for the mix of the individual vaccine vaccinations) served as controls. All groups of mice were then boosted with MVA (10^4^ pfu) on day 28. On day 40 spleens were harvested and splenocytes pooled from 5 mice per group were used in an IFN-γ ELISPOT (**A**) or IL-2 ELISPOT (**B**) assay with Gag, RT, Env and peptide H CD8^+^ T cell and CD4^+^ T cell peptides. Bars are the average and standard deviation of the average IFN-γ ELISPOT (A) or IL-2 ELISPOT responses for the indicated individual peptides for 3 separate experiments. Statistical significance (Student's t-test for means of unpaired data) of the mean IFN-γ ELISPOT or IL-2 ELISPOT responses for the individual peptides compared to that for the control is indicated.

The vaccine mix did prime the immune system for a rMVA boost (Figure 4A and B). Cumulative responses to Gag-, RT- and peptide H-specific CD8^+^ and Gag-, RT- and Env-specific CD4^+^ T cell peptides in the IFN-γ ELISPOT assay reached 1798±52 sfu/10^6^ splenocytes, approximately 3.5-fold (p<0.01) above that for responses to these peptides induced by the BCGpan-Control prime and rMVA boost (Figure 4A). Subdominant Gag- and RT-specific CD8^+^ T cell and Gag-, RT- and Env-specific CD4^+^ T cell responses contributed 66% to the cumulative response for a prime with the vaccine mix. Cumulative magnitudes of the IFN-γ ELISPOT peptide responses induced by the vaccine mix did not reach the sum of responses induced by the individual vaccines (Figure 4A). Priming with the individual rBCGpan-Gag, rBCGpan-RT or rBCGpan-gp120 vaccines induced cumulative IFN-γ ELISPOT CD8^+^ T cell and CD4^+^ T cell peptide responses of 1431±151 sfu/10^6^ splenocytes; 2109±185 sfu/10^6^ splenocytes; and 1173±94 sfu/10^6^ splenocytes respectively. These responses are between 3.5 and 5.2 fold (p<0.01) above that for a rBCGpan-Control prime (Figure 4A). Subdominant responses contributed 38%, 55% and 20% to the cumulative IFN-γ ELISPOT CD8^+^ T cell and CD4^+^ T cell peptide responses for the respective individual rBCGpan vaccine prime and rMVA boost. Thus it appears that the proportion of subdominant peptide-specific responses to the cumulative IFN-γ ELISPOT response can be increased from an average of 37±17% for an individual vaccine prime to 66% when a vaccine mix is used as the prime.

IL-2 ELISPOT assays indicated only HIV-specific CD4^+^ T cells produced IL-2 in response to a prime with either the vaccine mix or the individual vaccines (Figure 4B). Cumulative IL-2 ELISPOT responses to Gag, RT and Env CD4^+^ T cell peptides in response to a vaccine mix prime reached 356 sfu/10^6^ splenocytes which was 3-fold (p<0.05) above that for responses to these peptides induced by priming with the rBCGpan-Control. These frequencies of HIV-specific IL-2 producing cells were similar to that induced by a prime with the individual vaccines (Figure 4B).

### Th1 cytokine production

**Figure 5:**
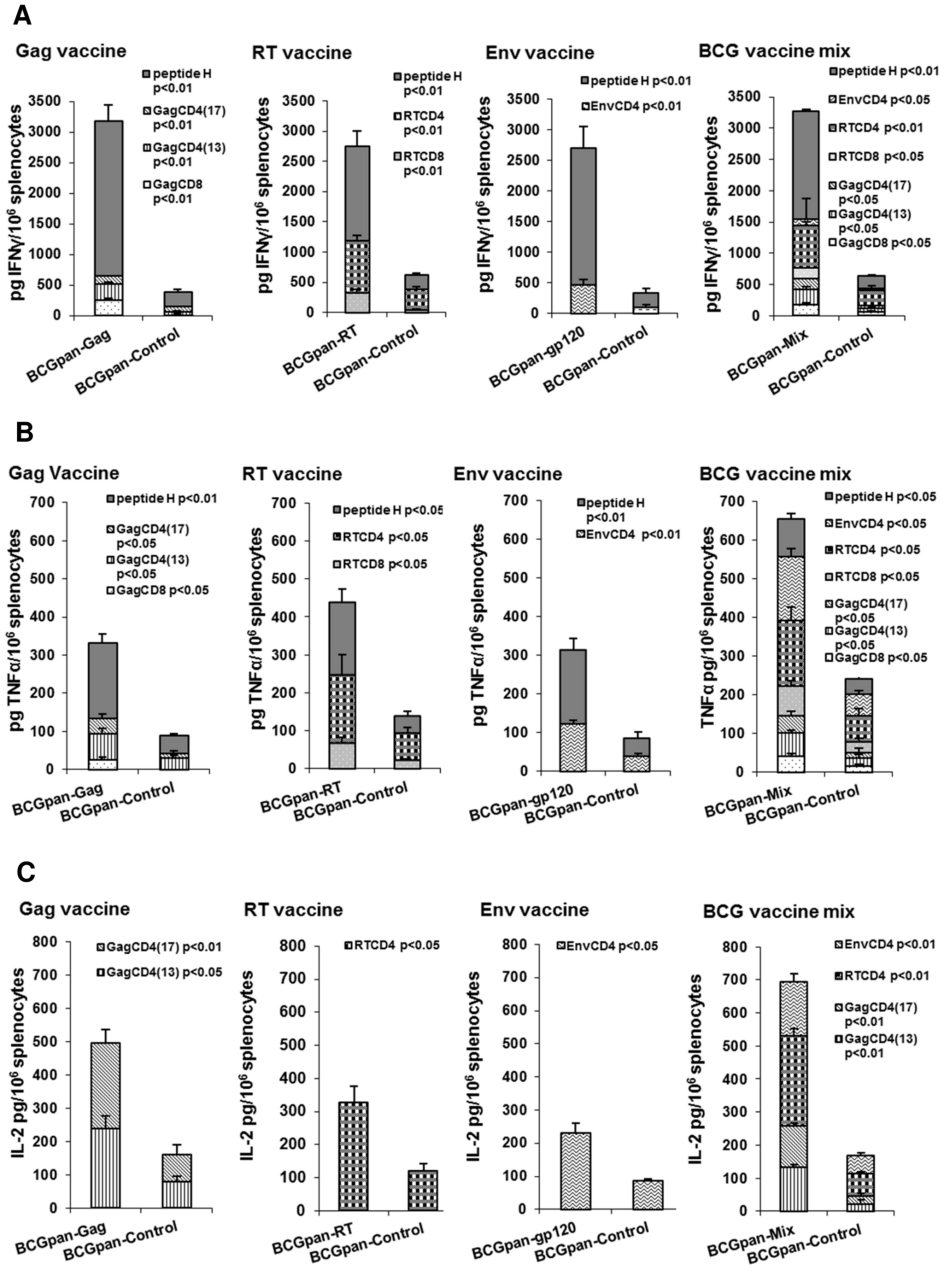
Th1 cytokines induced by a prime with the individual rBCG-HIV vaccines or a mix of the rBCG-HIV vaccines and rMVA boost. Groups of mice were primed with either the individual rBCGpan-Gag, rBCGpan-RT or rBCGpan-gp120 vaccines (10^7^ cfu) or a rBCG vaccine mix (3×10^7^ cfu) prepared by mixing 10^7^ cfu of the individual vaccines rBCGpan-Gag, rBCGpan-RT and rBCGpan-gp120. Groups of mice vaccinated with the BCGpan-Control vaccine (10^7^ cfu to act as control for the individual vaccine vaccinations or 3×10^7^ cfu to act as control for the mix of the individual vaccine vaccinations) served as controls. All groups of mice were then boosted with rMVA (10^4^ pfu) on day 28. On day 40 spleens were harvested and splenocytes pooled from 5 mice per group were stimulated with Gag, RT, Env and peptide H CD8^+^ T cell and CD4^+^ T cell peptides. Culture supernatants collected at 48 h were analyzed for IFN-γ (**A**), TNF-α (**B**) and IL-2 (**C**) using a cytokine bead array assay and flow cytometric analysis. Data was expressed as pg cytokine/10^6^ splenocytes. Bars are the average and standard deviation of the average cytokine released into the supernatant during stimulation with the indicated individual peptide for 3 separate experiments. Statistical significance (Student's t-test for means of unpaired data) of the mean cytokine response for the individual peptides compared to that for the control is indicated.

The Th1/Th2 bias of the immune response to a prime with the individual vaccines (10^7^ cfu) or a mix of the individual vaccines rBCGpan-Gag, rBCGpan-RT and rBCGpan-gp120 (3×10^7^ cfu) and rMVA boost was investigated (Figure 5). rBCGpan-Control (10^7^ or 3×10^7^ cfu) was used as the control vaccine prime. Th1 and Th2 cytokines were quantified in the supernatant collected from splenocytes cultured with Gag, RT and peptide H CD8^+^ T cell peptides and Gag, RT and Env CD4^+^ T cell peptides. No IL-10 or IL-4 could be detected in the supernatants for a prime with the vaccine mix and rMVA boost as well as a prime with the individual vaccines and rMVA boost.

A prime with the vaccine mix and rMVA boost induced peptide-specific IFN-γ and TNF-α in the culture supernatants in response to stimulation with Gag, RT and peptide H CD8^+^ T cell and Gag, RT and Env CD4^+^ T cell peptides. These individual peptide-specific IFN-γ and TNF-α levels were significantly different (p<0.01 or p<0.05) from the rBCGpan-Control prime and rMVA boost (Figure 5). Cumulative HIV peptide-specific IFN-γ levels of 3269±198 pg/10^6^ splenocytes and TNF-α of 655±150 pg/10^6^ splenocytes were measured for a vaccine mix prime and rMVA boost which were 5 fold (p<0.01) and 3 fold (p<0.05) higher respectively than for a rBCGpan-Control prime and rMVA boost (Figure 5A and B). These IFN-γ and TNF-α levels, for a rBCGpan-Control prime and rMVA boost, were not significantly different from a rMVA vaccination alone. For a vaccine mix prime and rMVA boost 48% of the total IFN-γ was produced by subdominant peptide-specific CD8^+^ and CD4^+^ T cells. The total TNF-α production was comprised of 85% from subdominant peptide-specific CD8^+^ and CD4^+^ T cells. The cumulative magnitudes of CD8^+^ T cell and CD4^+^ T cell peptide-specific IFN-γ and TNF-α for a vaccine mix prime and rMVA boost were lower than the sum of these responses for a prime with the individual vaccines and rMVA boost (Figure 5A and B). A prime with the individual rBCGpan-Gag, rBCGpan-RT or rBCGpan-gp120 vaccines and rMVA boost induced cumulative HIV-peptide specific IFN-γ magnitudes of 3182±102 pg/10^6^ splenocytes, 2745±120 pg/10^6^ splenocytes and 2713±110 pg/10^6^ splenocytes that were 4.5 to 8 fold (p<0.01) higher respectively than a rBCGpan-Control prime and rMVA boost (Figure 5A). Subdominant peptide-specific CD8^+^ and CD4^+^ T cells contributed 21%, 43% and 18% to the total IFN-γ production for the respective individual rBCGpan vaccine prime and rMVA boost (Figure 5A).

Cumulative peptide-specific TNF-α levels of 333±28 pg/10^6^ splenocytes, 440±32 pg/10^6^ splenocytes and 315±15 pg/10^6^ splenocytes induced by a prime with the individual rBCGpan-Gag, rBCGpan-RT or rBCGpan-gp120 vaccines and rMVA boost, were at least 3 fold (p<0.01) greater than those from a rBCGpan-Control prime and rMVA boost (Figure 5B). Subdominant peptide-specific CD8^+^ and CD4^+^ T cells contributed 40%, 57% and 40% to the total TNF-α production for the respective individual rBCGpan vaccine prime and rMVA boost (Figure 5B).

Thus it appears that when compared to a prime with the individual vaccines, a vaccine mix prime and rMVA boost induces increased subdominant peptide-specific IFN-γ and TNF-α production. The proportion of subdominant peptide-specific IFN-γ to the total peptide-specific IFN-γ response increased from an average of 27±13% for an individual vaccine prime to 48% using a vaccine mix as the prime. The contribution of subdominant peptide-specific TNF-α to the total peptide-specific TNF-α response increased from an average of 45±9% induced by an individual vaccine prime to 85% using a vaccine mix as the prime.

No IL-2 production by peptide-specific CD8^+^ T cells could be detected for any of the vaccine regimens. A prime with the vaccine mix and rMVA boost induced peptide-specific IL-2 levels in the culture supernatants in response to stimulation with Gag, RT and Env CD4^+^ T cell peptides which were significantly different (p<0.01,) from the individual peptide-specific IL-2 levels in the culture supernatants from the rBCGpan-Control prime and rMVA boost (Figure 5C). Cumulative HIV CD4^+^ T cell peptide-specific IL-2 levels of 695±22 pg/10^6^ splenocytes were measured for a prime with the vaccine mix and rMVA boost, which were four fold higher (p<0.01) than those produced by a rBCGpan-Control prime and rMVA boost (Figure 5C). The levels of HIV peptide-specific IL-2 induced by rMVA vaccination were not significantly different to that of a rBCGpan-Control prime and rMVA boost. The cumulative magnitudes of CD4^+^ T cell peptide-specific IL-2 for a vaccine mix prime and rMVA boost were lower than the sum of these responses for a prime with the individual vaccine and rMVA boost (Figure 5C). A prime with the individual rBCGpan-Gag, rBCGpan-RT or rBCGpan-gp120 vaccines and rMVA boost induced cumulative CD4^+^ T cell peptide-specific IL-2 levels of 496±32 pg/10^6^ splenocytes, 328±48 pg/10^6^ splenocytes and 230±30 pg/10^6^ splenocytes that were 3 fold higher (p<0.05) than for a rBCGpan-Control prime and rMVA boost (Figure 5C).

## Discussion

Studies evaluating CD8^+^ T cell priming by rBCG indicate highly functional memory T cells which are able to proliferate and produce cytokines are generated to foreign expressed proteins [26]–[29]. Several factors appear to participate in this differentiation to antigen specific cells with a memory phenotype. Slow BCG replication rates within phagosomes and limited levels of antigen expression that peaks in the second weak after infection are factors that participate in this process [5], [30]. A previous study of ours showed that a rBCGpan-Gag prime and Gag VLPs boost vaccine regimen is highly immunogenic and induces a broad and polyfunctional central memory T cell response in baboons [10]. Other studies by Hanke *et* *al*. have also demonstrated rBCG effectively primes the immune system for a rMVA boost to elicit HIV-specific T cells [6]–[8], [31]. Thus in this study a rBCG vaccine prime and rMVA vaccine boost regimen was chosen to investigate maximum immune responses to rBCG vaccines expressing various HIV proteins. The rMVA chosen for the study, SAAVI MVA-C, which is in phase I clinical trials [17], expresses proteins identical to that expressed by the individual rBCG vaccines tested in this study.

Analysis of genetic stability is an important aspect of characterization of candidate vaccines. In this study we were able to generate genetically stable (*in vivo* and *in vitro*) rBCG and rBCGΔ*panCD* expressing Gag, RT or a truncated Env (Gp120). No stable recombinants expressing the HIV-1 *gp150* gene could be obtained. Instability of rBCG expressing HIV or SIV antigens has been reported by a number of different groups. Cayabyab *et* *al*. [4] reported that they were unable to obtain any rBCG expressing SIV Gp160 and rBCG expressing SIV Gp120 were unstable. Two other groups also found that rBCG expressing truncated *env* genes utilising the strong *hsp60* promoter were unstable, whereas one of the groups could obtain stable rBCG expressing Gp120 when they used the weaker α-antigen promoter for expression [32], [33]. As suggested in a previous paper, in which we were able to improve the stability of rBCG expressing HIV-1 Gag [5], the genetic stability of our vaccines could be due to two factors: (i) the use of the *mtrA* promoter which expresses weakly *in vitro* but is strongly up-regulated after uptake by phagocytosing cells and (ii) the fusion of the HIV antigen to the 19kDa leader sequence which transports the protein to the surface of the mycobacteria, thus removing a possibly toxic antigen from the mycobacterial cell.

Experiments using two different assays, the IFN-γ ELISPOT assay and enumeration of pentameric MHC class I-peptide-specific reactive CD8^+^ T cells, were performed to evaluate the influence of the vector, BCG or BCGΔ*panCD*, on immune responses to expressed Gag and RT. An overall conclusion from the data obtained from the two different assays is that using BCGΔ*panCD* rather than BCG as a vector is beneficial for the induction of higher frequencies of total HIV-specific T cells and increased numbers of T cells specific to the subdominant Gag and RT epitopes. An interesting observation using the IFN-γ ELISPOT assay is that the magnitude of peptide H-specific CD8^+^ T cell responses (when attached to the Gag or RT protein) were not affected by the choice of vector. In contrast using the direct enumeration method, the magnitude of peptide H-specific CD8^+^ T cell frequencies (when attached to the Gag protein), were significantly higher with BCGΔ*panCD* as the vector with the lower BCGpan-Gag vaccine dose eliciting higher peptide H-specific CD8^+^ T cells frequencies than the higher dose. Although this was not seen for the frequencies of peptide H attached to the RT protein frequencies of RT CD8^+^ T cells when directly enumerated were significantly higher when RT was expressed by BCGΔ*panCD* rather than BCG. This was not detectable using the IFN-γ ELISPOT assay. An underlying difference between the assays is that the IFN-γ ELISPOT assay detects peptide-specific cells that respond during *in vitro* peptide stimulation. This recall response is not operative in the direct enumeration assay. It is possible that by directly enumerating peptide-specific responses, differences are more easily detected as inhibitory and/or stimulatory products generated during the culture conditions may influence the magnitude of responses. In addition the finding that peptide H-specific CD8^+^ T cell frequencies (when attached to the Gag protein) are greater for lower vaccine doses suggests *in vivo* inflammatory conditions may affect the development of immune responses to Gag, but possibly not affect RT immune responses. We have shown previously [5] that BCGΔ*panCD* is less inflammatory than BCG which would support lower inflammatory conditions influencing higher magnitudes of CD8^+^ T cells frequencies (peptide H-specific CD8^+^ T cells for the BCGpan-Gag vaccine and RT-specific CD8^+^ T cells frequencies for the BCGpan-RT vaccine) using the BCGΔ*panCD* vector.

The reason for the broader response seen with the use of the pantothenate auxotroph as a vector over that of wild type BCG is not clear, however it may be related to the fact that cellular responses to immunodominant epitopes can limit responses to subdominant epitopes [34] and the differences in *in vivo* inflammatory conditions generated by BCG and BCGΔ*panCD*. Pantothenate is required for protection of bacteria from oxidative stress. In addition pantothenate is a key precursor of coenzyme A and acyl carrier proteins that are essential for many intracellular processes including fatty acid metabolism. Lack of pantothenate could result in an alteration in bacterial lipid metabolism and exposure to oxidative stresses which may disrupt the survival of the bacteria in the phagosome and promote phagosome maturation and subsequently CD8^+^ T cell responses. This may operate in conjunction with the fusion of the 19 kDa lipoprotein signal sequence to the HIV proteins that allows acylation of the signal sequence, MHC class I presentation and interaction of the antigen with Toll-like receptor 2, which are processes that promote induction of HIV-specific CD8^+^ T cells [35]. A recent study comparing Gag-specific responses when expressed by wild type BCG and the Δ*panCD* auxotroph indicated that while clearance of the bacteria occurred at the same rate, there was far less tissue damage, less inflammation and less granuloma formation with the Δ*panCD* auxotroph which is a favourable factor for the use of the auxotroph [5]. In this study, higher inflammation generated by the wild type strain of BCG may also have inhibited the development of the response to the subdominant epitopes after the rMVA boost. IFN-γ ELISPOT results indicated that delaying the time of the rMVA booster vaccine to day 56 increased the immune response to subdominant epitopes (data not shown), which probably arises due to a time dependent decrease in the overall activation of the immune system by innate BCG immune responses. It is also possible that use of the BCGΔ*panCD* vector results in higher and more sustained production of the expressed proteins and thus development of the immune response to subdominant epitopes.

The ability of vaccines to induce responses to multiple HIV proteins is desirable with the broad immune responses increasing the possibility of overcoming immune escape. HIV-specific CD4^+^ and CD8^+^ T cells recognising HIV-dominant and subdominant epitopes were detected after a prime with a mix of BCGΔ*panCD* expressing either Gag, RT or Gp120 proteins and a boost with rMVA. Higher inflammatory responses generated when using a dose of 3×10^7^ cfu may account for the lower total IFN-γ ELISPOT responses achieved with the vaccine mix when compared to the sum of the responses to the individual vaccines used at a dose of 10^7^ cfu. It is also possible that the simultaneous presentation of HIV antigens by the vaccine mix and thus a presentation of a broader repertoire of HIV epitopes than with an individual vaccine, contributes to the observed lower immune responses induced by the vaccine mix. However it is of importance to note that the vaccine mix induced a broad T cell immune response which is a desirable attribute of a vaccine. High levels of CD8^+^ and CD4^+^ T cells recognising both subdominant and dominant epitopes in Gag, RT and Gp120 that produced IFN-γ and TNF-α as well as CD4^+^ T cells that produced IL-2 were generated. Vaccine-specific CD4^+^ T cells producing IL-2 are a desirable outcome of vaccination as these cells are now understood to be preserved in response to infection and to participate in suppression of viremia levels post infection [36]–[38]. In this study CD4^+^ cell produced IL-2 production may have provided help for the induction of the HIV-specific CD8^+^ T cells.

A number of different studies have shown that recombinant mycobacteria expressing HIV-1 antigens prime the immune system for a heterologous booster vaccine. Macaques vaccinated with rBCG-SIVgag and boosted with a replication deficient vaccinia virus expressing SIV Gag were protected against a mucosal challenge with pathogenic SHIV [9]. In another study monkeys vaccinated with BCG expressing HIV-1 Gag, Pol and a truncated Env elicited a robust, polyfunctional CD8^+^ T cell response following a boost with Ad5 [4]. Tomas Hanke's group have shown, in a number of different studies, that a BCG.HIVA prime followed by a boost with a viral vector expressing the HIVA immunogen, can induce stable, robust, HIV-specific T cell responses [6]–[8], [31]. The rBCG vaccines utilised in this study were able to prime the immune system for a boost with rMVA expressing matching antigens, inducing HIV-specific T cells to both dominant and subdominant epitopes in the individual proteins in BALB/c mice, when used as individual vaccines or in a mix. The expressed HIV proteins contained BALB/c mouse MHC-I H-2K^d^ and H-2D^d^ as well as MHC-II restricted epitopes which allowed detection of epitopic breadth of vaccine elicited T cells to be determined. Further studies using these vaccines are currently being carried out in non-human primates.

Although the induction of HIV-specific polyfunctional memory cells was not enumerated in this study data from other studies has indicated that BCG shows great promise as an HIV vaccine which can elicit a broad, robust, polyfunctional T cell response when used as a prime with a heterologous boost. In addition it is cheap to produce, has a good safety record and could be used as a combined vaccine against both HIV and TB. However, BCG can cause disseminated disease in immunocompromised individuals. Thus as BCGΔ*panCD* is safer than wild type BCG in SCID mice, causes less tissue damage in mice, shows attenuated growth *in vivo* and gives a broad HIV-specific T cell response to the insert when used as a vaccine vector without compromise of BCG-specific immune responses (data not shown), BCGΔ*panCD* may be a suitable HIV vaccine vector for use in immunocompromised individuals.
