# Correction: Priming with Recombinant Auxotrophic BCG Expressing HIV-1 Gag, RT and Gp120 and Boosting with Recombinant MVA Induces a Robust T Cell Response in Mice

**DOI:** 10.1371/annotation/4f08219c-2d7b-4309-8351-d3fe2378993f

**Published:** 2013-09-06

**Authors:** Rosamund Chapman, Helen Stutz, William Jacobs, Enid Shephard, Anna-Lise Williamson

There were errors in the affiliation given for authors. The correct list of affiliations is available below.

^1^Institute of Infectious Disease and Molecular Medicine, University of Cape Town, Cape Town, South Africa,

^2^Division of Medical Virology, Department of Clinical Laboratory Science, University of Cape Town, Cape Town, South Africa,

^3^Department of Microbiology & Immunology, Albert Einstein College of Medicine, Bronx, New York, USA,

^4^Department of Medicine, Faculty of Health Sciences, University of Cape Town, Cape Town, South Africa,

^5^Medical Research Council, Cape Town, South Africa,

^6^National Health Laboratory Service, Cape Town, South Africa. 

